# The Mental Health Implications of Living in the Shadows: The Lived Experience and Coping Strategies of Undocumented African Migrant Women

**DOI:** 10.3390/bs9120127

**Published:** 2019-11-26

**Authors:** Oluwatoyin Olukotun, Kaboni Gondwe, Lucy Mkandawire-Valhmu

**Affiliations:** College of Nursing, University of Wisconsin–Milwaukee, Milwaukee, WI 53201, USA; gondwe@uwm.edu (K.G.); mkandawi@uwm.edu (L.M.-V.)

**Keywords:** immigrants, mental health, undocumented immigrants, stressors, migration

## Abstract

In the United States, undocumented immigrants often encounter complex challenges that impact their emotional well-being. Existing literature has primarily focused on Latino immigrants. Thus, little is known about the mental health needs of undocumented African immigrant women. To address this gap, we examined the stressors, mental health concerns and coping strategies of undocumented African migrant women in the United States. This qualitative study used a postcolonial feminist framework approach. Twenty-four undocumented African migrant women were interviewed, and data were analyzed using thematic analysis. Findings showed that the women dealt with complex stressors created by the sociopolitical environment. These stressors contributed to feelings of depression and anxiety which they coped with using social support and religion. The results uncover the need for culturally relevant tools for screening and addressing the mental health needs of undocumented women and increased awareness amongst healthcare providers on how social context and policies adversely impact the mental health of marginalized groups. Lastly, at a structural level, the need for policy and social change that fosters an inclusive and safe environment for undocumented persons.

## 1. Introduction

The Black, African immigrant population in the United States (U.S.) nearly tripled between 2000 and 2013 from almost half a million to nearly 1.5 million [1] with over 60% of the population originating from Nigeria, Ethiopia, Ghana, Kenya, Somalia, and Liberia [2]. Although there are a variety of avenues for immigrating to the U.S., African immigrants seeking permanent residence typically gain admission to the U.S. either as refugees or as recipients of the diversity lottery program visa. Alternative modes of entry into the U.S. for most African immigrants include temporary non-immigrant visas such as student visas, or through U.S. permanent residence visas conferred through family reunification or employment provisions [2]. However, despite the outlined immigration paths, due to the per-country quotas enforced in U.S. immigration law, there are notable disparate effects that limit authorized immigration from African countries [3,4]. Hence, African immigrants may opt for other means to long-term residence in the U.S. (such as an overstay of a temporary visa) which inadvertently results in a precarious migrant status. Migrants with precarious statuses, such as those who are undocumented, comprise 13% of the U.S. African immigrant population [5].

According to the U.S. Department of Health and Human Services, the term undocumented immigrant refers to “individuals who entered as temporary residents and overstayed their visas, or are engaged in activities forbidden by their visa, or who entered without a visa” [6]. Changing policies and practices in the past 10 years have magnified the challenges associated with being undocumented in the U.S., making it challenging for undocumented immigrants to access health and social services [7,8]. Additionally, there has been a drastic rise in deportation rates in the past 20 years [9]. More recently, the challenges associated with being undocumented have been compounded by an increased visibility and normalization of xenophobic, racist and nativist sentiments in the U.S. [10,11,12]. Consequently, undocumented immigrants must cope with a variety of complex stressors resulting from restrictive and punitive policies as well as an increasingly hostile environment [13,14]. Yet, there is a paucity of evidence on how the current sociopolitical environment created by restrictive policies and hostile rhetoric might impact the psychological well-being of African undocumented immigrants. To address this gap, this paper reports focused findings on the stressors, mental health concerns and coping strategies for 24 undocumented, Black African migrant women.

A few studies have examined mental health status of immigrant populations. Existing literature indicates that immigrants are exposed to specific risks factors that impacts their mental health at different phases of the migration process [15,16,17]. African migrants, for instance, often migrate to escape conflict, political instability, persecution and/or economic insecurity [18]. The resultant pre-migration traumatization and violence has implications for their mental health [19]. For undocumented immigrants, the mental health effect of premigration stressors are further compounded by distinct post-migration stressors including economic difficulties, separation from family, fear of deportation and detection, exploitation, vulnerability, fewer familial networks, and language barriers [20].

A few recent studies have specifically focused on mental health outcomes for undocumented immigrants, mostly in immigrants from Latin and South America. Hacker et al. [21] revealed that both documented and undocumented immigrants reported high levels of stress, anxiety and hopelessness, which had a negative impact on their emotional well-being. For undocumented immigrants, their feelings of stress, anxiety and hopelessness were related to the constant fear of deportation while documented participants were more concerned about the welfare of their family living in the U.S. The differences in the drivers of psychological distress for undocumented immigrants illustrate the unique challenges that undocumented immigrants face. Other studies have identified that undocumented immigrants were found to be at higher risk of depressive symptoms, post-traumatic stress disorder, anxiety, and reported that discrimination and undocumented status affected their mental well-being resulting in weight gain/loss, insomnia, anxiety, depression, substance abuse and fear [22,23,24,25]. Additionally, anxiety and depressive symptoms have been found to be directly related to immigration status [26].

The role of discrimination as a stressor is particularly relevant for Black, African immigrants who often leave their racially homogenous birth countries to a racialized society where racial and ethnic minorities experience marginalization [27,28]. The manifestation of social marginalization experienced by Black, African immigrants is evidenced by documented reports of discrimination and racism upon arrival to the U.S. [29,30]. The implications of racial discrimination for immigrants are grave, as racism and discrimination has been linked to worsening health in immigrant populations [31,32]. Though African immigrants arrive to their Western host countries being relatively healthy, a phenomenon termed “the healthy immigrant effect”, this advantage erodes with time. This is notable in mental health outcomes of immigrants for whom rates of mental health conditions increase over time to match the general population [33] as well as for undocumented immigrants who have higher rates of anxiety, panic disorder and depression compared to the general population [34]. This change has been attributed to a combination of factors including experiences of discrimination, which have been identified as a significant risk factor and a driver of psychological distress in immigrants [22,32,35].

Despite the established need for mental health care services for immigrants, there are significant cognitive and structural barriers to seeking care. A study of Latino undocumented immigrants found that even though immigrants who were undocumented had a high burden of mental health symptoms and psychosocial stressors, they were less likely to utilize mental health care [36]. Generally, African immigrants are also less likely to utilize mental health services when experiencing psychological distress. This is in part a result of the stigma associated with mental health symptoms [19]. Other barriers to accessing mental health services for immigrants include beliefs about mental illness, distrust of providers, cost of care, language barrier and other health demands [37]. Though undocumented African immigrants might not seek formal mental health care, prior work has found that immigrants develop adaptive coping strategies to deal with their stressors and psychological distress. Literature on immigrant coping strategies have identified social support, participating in meaningful activities, religious coping and belonging to a religious community as helpful strategies [38,39]. A study on undocumented Hispanic immigrants, however, revealed that problem-focused coping strategies such as prayer and meditation might exacerbate feeling of psychological distress because undocumented immigrants experience complex, structural issues that are often beyond their ability to solve [40]. It is, therefore, essential that we understand how these complex realities impact mental health and how undocumented women specifically, effectively cope. Existing literature on stressors and mental health symptoms largely focuses on Latino immigrants in the U.S. Additionally, very few studies have simultaneously considered the stressors that undocumented women experience, the mental health implications and how they cope with them. To address this gap, using a postcolonial feminist lens that foregrounds how context shapes women’s experience, this paper reports on the stressors, mental health concerns and coping strategies for undocumented, Black African migrant women in the U.S.

### Theoretical Framework

In an effort to elucidate information on the interrelatedness of women’s context and experiences, and to highlight how women act as agents within their given context, this study was undergirded by a postcolonial feminist framework. Postcolonial feminism represents a paradigm shift from the narrative of cultural hybridity that historically typified Western hegemonic discourse on women of color. Postcolonial feminism asserts that the experiences of women of color are influenced by larger sociopolitical processes acknowledging that women are situated in distinct spaces, and their realities are shaped by these sociopolitical processes [41]. Postcolonial feminism provides an analytic lens that allows for an examination of the experiences of undocumented African migrant women in the U.S., taking into account the impact of contextual factors such as racism, xenophobia, and attitudes toward immigrants, policies and other sociopolitical factors in their lives.

In attempting to change the current hegemonic narrative of African women, postcolonial feminism is purposeful about critiquing traditional methods of knowledge production by challenging the positivist notion that knowledge can be objective and value-free. Rather, postcolonial feminism emphasizes the idea that knowledge is socially constructed, political and value laden. Mohanty asserts that knowledge production is “a mode of intervention into particular hegemonic discourses” with its practices embedded in existing power hierarchies therefore, the concept of “apolitical scholarship” is nonexistent [42]. Acknowledging the political implications of scholarship means naming the complex forces in action specifically in relation to third world women but also being deliberative in creating a space where women’s voices can be heard and can inform health and social policy. In essence, postcolonial feminist researchers should not perceive themselves as having the power to emancipate individuals who they perceive as being powerless. In contrast, a postcolonial feminist lens demands that researchers recognize the human agency and capabilities of marginalized people in initiating and creating change. Emancipation occurs when scholars work in partnership with individuals who inhabit marginalized spaces. It is through this engagement and mutual and dialectic relationship centered around learning that true emancipation occurs [43,44]. Hence, beyond generating knowledge, our aim in this paper is to center women’s narratives as the foundation for recommendations aimed at driving social, practice and policy changes.

## 2. Materials and Methods

This descriptive, qualitative study involved data collection using semi-structured interviews with open-ended questions. Purposive sampling was utilized to recruit an initial sample of women through collaboration with local churches and community leaders. Concurrently, snowballing was employed to recruit additional participants for the study. To be eligible for the study, women had to self-identify as being undocumented, an African immigrant, aged 18 years or older, and be fluent in English or French. For the purpose of this study, having an undocumented status was defined as not having authorized or legally recognized presence in the U.S. at the time of the interview. A total of 24 women were recruited and interviewed for the study. Nineteen interviews were conducted in person, while five were conducted over the phone. Interviews commenced with participants being asked demographic information such as their age, country of birth, marital status, educational level, employment status and annual income. To elicit information regarding their mental health concerns and coping, women were asked to respond to the following questions—“do you have any concerns about your emotional well-being? What do you do when you are feeling stressed? How does it feel being an undocumented African woman in the United States? What challenges do you face?” (Appendix A). The women interviewed received a $30 gift card upon completion of the interview. Each interview was audio-recorded and the average duration for the interview was one hour. The interviews were transcribed and analyzed using the tenets of thematic analysis.

### 2.1. Ethical Consideration

Ethical approval to conduct the study was obtained from the University of Wisconsin–Milwaukee’s Institutional Review Board. Prior to conducting the interview, the researcher reviewed a study informational sheet with the women that outlined the study’s procedures, risks and informed women of their ability to stop the interview at any time. After reviewing the informational sheet, women were given the opportunity to ask questions. Maintaining women’s anonymity was critical during the study considering the sensitive nature of their undocumented status and the potential legal implications of discovery. Verbal consent was thus obtained from the women.

### 2.2. Sample

All the participants interviewed identified as Black, African migrant women from Eastern, Southern or Western Africa (to preserve participants’ confidentiality, any reference to the participant’s native country has been omitted from the paper). The average age of the participants was 36 years. The majority (n = 21) of the participants lived in Midwestern U.S. The other women interviewed lived in the Southern or Eastern region of the country. Though study eligibility was open to both French-speaking and English-speaking women, all women who participated in the study were English-speaking. Thirteen women interviewed reported an annual household income of $19,999 or less. The average length of stay in the U.S. for women in the sample was 12 years. Nine women worked full time; one worked part time; seven reported having unstable jobs with irregular hours and seven were unemployed. The women mostly had an associate degree or higher, had a least one child and were not married at the time of their interview (Table 1).

### 2.3. Data Analysis

Interviews conducted were audio-recorded and analyzed using thematic analysis. The goal of data analysis was three-fold: first, to identify themes pertaining to the stressors, concerns about emotional well-being and coping strategies. Second, to situate women’s experiences within their contexts and lastly, to foreground women’s realities acting as agents. First, the researcher reviewed the transcripts to check for accuracy. Then, each transcript was re-read with the intention of identifying themes and patterns across the participant’s experiences. During the coding process, the researcher read each transcript and highlighted texts that were pertinent to the research questions and assigned codes to the text. The coding process was repeated for each transcript until they were reduced into themes and subthemes. Analysis of the interviews was a collaborative process that involved the researcher and her research mentor (L.M.-V) having discussions about emergent themes and the study of conceptual framework. The data reported in this manuscript was condensed into three themes, which will be discussed.

### 2.4. Trustworthiness

Informed by a postcolonial feminist lens, several strategies were employed to ensure trustworthiness of the data. To enhance confirmability, the researcher kept a reflexive journal throughout the research process. Reflections during the research process focused on acknowledgement of social positioning, bias and values. First, critical reflexivity required of the researcher ensures that the individual become conscious of how their positionality and subjective experience influences the research process. More importantly, it forces the researcher to maintain a conscious awareness of the importance of preventing the reproduction of power structures, while also acknowledging one’s privilege and accountability in creating unjust conditions [41,44]. The journal included information such as the researcher’s thoughts and personal reactions to women’s narratives. To enhance credibility, member checking was also employed by discussing the researcher’s interpretations obtained after data analysis with three participants to verify accuracy. The researcher also verified accuracy of interpretations during each interview by summarizing and paraphrasing women’s responses and clarifying these with the women. This was essential, as postcolonial feminist research encourages a collaborative inquiry directed by both the research and the participants. Further, the use of participants’ insight and critique during the analysis was a deliberate effort to ensure that the women’s authentic narratives and stories were properly deconstructed and articulated [41]. Transferability in this study will be because detailed demographic data of the participants has been provided alongside other data that help contextualize women’s narratives. Dependability was established using audit trail.

## 3. Results

This manuscript details our findings on the stressors undocumented African migrant women experience, the implications of these stressors on their emotional well-being and their coping strategies. Broadly, the experiences that women described were situated in a hostile environment, where a culmination of policies and societal attitudes had considerable implications for their daily lives and their emotional well-being. Three major themes were identified: experiencing stressors, mental health implications, and coping strategies. The stressors women reported included economic vulnerability, uncertainty and isolation. This resulted in women feeling sad and experiencing increased anxiety. Consequently, they turned to their social networks and religion to cope.

### 3.1. Experiencing Stressors

Women reported experiencing multiple, complex stressors. The stressors women described can be classified under three subthemes: vulnerability, feeling stuck, feeling alone. Their experiences of discrimination are described in detail in a different manuscript but will be discussed briefly in this paper.

#### 3.1.1. Vulnerability

Generally, the women interviewed indicated that they felt economically vulnerable. This was particularly true for women who were unemployed, employed casually or had low-income jobs. Many women were forced to work low-income jobs as a result of their immigration status and not having the proper documents that enabled them to work. Consequently, women were generally under-employed. For a few women interviewed, their current jobs were a drastic change from the professional jobs they worked in their home countries. A salient example of this was a participant with a Bachelor’s degree who had years of experience working as a human resources manager in her home country. As an undocumented immigrant in the U.S., she worked as a home care aide and a waitress for an annual income of less than $10,000.

Women’s feeling of economic vulnerability resulted in them being vulnerable to exploitation. Over half of the women interviewed believed that they were underpaid in their jobs but could not leave because they had limited employment options. Women were thus often unhappy with their employment situation. For women who had no work authorization and were not employed, they often relied on a spouse, relative and/or friend for their financial needs. This enhanced their feelings of vulnerability as they were then dependent and concerned about having to be dependent for the rest of their lives. One participant expressed her concerns about her cousin who had been housing and supporting her financially since she arrived to the U.S. She stated:

*“Sometimes I just feel down when I think about my life. I feel like my cousin is getting tired of hosting me. She acts irritated with some things I do. She wasn’t like that before. I don’t blame her … she has tried a lot. I’m hoping God will make a way for me to file my papers. I pray about it a lot. Right now, I am scared to go out sometimes. I have heard stories of immigration picking people up at the store and their jobs. It’s very scary.”*

This participant expressed concerns about becoming a burden to her cousin. Her situation was relatively common, as eight of the women interviewed reported a living situation where they were reliant on someone else for accommodation. Another participant had experienced being asked to find alternative living arrangements by a trusted friend who was housing her and had promised to host her upon her arrival to the U.S.

Women who were employed but not authorized recognized the precarious nature of their employment. Their employment status was dependent on the “good-will” of the employer who knew that they were not authorized to work. This meant that women’s employment arrangements were not permanent or binding and in essence, and they were at the mercy of their employer. Consequently, 11 women reported working at jobs where they felt exploited and treated unfairly. Labor exploitation occurred in the form of women being required to work long hours, not getting work breaks, being underpaid, being overworked, unsafe work conditions or experiences of verbal abuse from employers. One participant who worked as a live-in caregiver expressed concern about being vulnerable to physical harm perpetrated by her employer. She was concerned that due to the nature of her job where she lived in people’s home, she could be harmed and “disappear”, and no one would know what happened to her. This concern comes as a result of stories she had heard about live-in caregivers being mistreated, abused and exploited by their employers.

#### 3.1.2. Feeling Stuck

In addition to economic vulnerability, women described uncertainty as causing them to also feel vulnerable. Though participants reported feeling a long-standing sense of uncertainty, their concerns where heightened by the current sociopolitical climate, which they perceived as being hostile. This was particularly true for women who were out of status and had no immigration proceeding pending. Women spoke of other undocumented persons in their communities being detained and deported, and this worried them. One participant shared her perceptions of the shift in climate between the previous political administration and the current one stating:

*“I feel ... I would say things are worse. Because we have a president who seems to be anti-immigrant and pro-deportation. Even though with President Obama everything wasn’t perfect either but at least he did DACA but a lot of people were covered under that.”*

Women were uncertain about whether they would be detected, what their future held and how changing policies and practices would impact them. Consequently, it was challenging for women to plan their lives or make future plans. One participant likened her situation to “feeling stuck”. Several women stated that whenever they could, they tried to continue on with their lives as though immigration status was not a factor. However, continuing on with their lives often meant that women had to re-conceptualize their aspirations. This meant enrolling in a local community college to pursue a degree, instead of a university, which they would have preferred and working at a job for which they were overqualified. To the best of their ability, women still attempted to maintain some degree of normalcy by proceeding to have children and attain personal milestones. However, they acknowledged that ignoring the constraints that their immigration status placed on their choices was not an option, as their status dictated the resources and services they could access. Even when they attempted to continue with their lives, their immigration status and its legal implications made it challenging for them to feel at peace, particularly considering the current sociopolitical climate.

Uncertainty was also related to the notion that most women interviewed were unsure when their situation would improve. Many women were hopeful that they would be able to change their immigration status at some point but they were uncertain as to when that would happen. They were also uncertain of how they would be able to attain this status change. Consequently, they had to cope with their current situation indefinitely. This realization was concerning and saddening for women.

#### 3.1.3. Feeling Alone

Despite being connected to a social network in the form of friends and relatives within their communities and in their home countries, women reported feelings of isolation. Women’s isolation was often centered on the fact that they felt alone in their experience as undocumented women. Feelings of isolation stemmed from the idea that most people “did not understand” the challenges they faced. One participant stated:

*“Like where do I belong. Here I am, in between ... I am black, immigrant, I am undocumented. The black community does not really address immigration. They focus more on Black American issues. When you go to immigrant spaces, a lot of those organization are mostly geared toward Latino immigrants, not really black people. You don’t really fit in there either. So it’s like, you’re a double minority on top everything. So it’s hard because I always feel othered. I think I have many different identities. In my day to day life I feel very othered.”*

This sentiment of not “fitting in” anywhere was worsened by women’s perception of not being able to rely on immigrants from their home country. Several participants felt that immigrants from their country intentionally withheld information from them about how to navigate the U.S. system. Women believed that the lack of collegiality and community among immigrants from their home country was because settled immigrants preferred that newly arrived immigrants experience the same painful and challenging process of integration as they did. In order to gain information about how to survive as immigrants, women thus sometimes had to reach out to networks of immigrants from varying backgrounds or figure out how to find information independently.

Women also felt isolated because they could not travel back to their country of origin to visit their loved ones given their undocumented status and the fact that the likelihood of being able to return to the U.S. should they exit the country was very slim. Consequently, several participants had not seen their family for years. For example, one of the women interviewed who was awaiting a decision on a petition for asylum had come to the U.S. without any relatives, and had not seen her family for 10 years. Women’s identity as undocumented immigrants was thus also isolating because they felt that they could not rely on just anyone for emotional support. They had to be cautious about who they disclosed their status to. Finding a trusted person or reliable outlet or support was not an easy feat. One participant shared her experience as follows:

*“It’s very easy to feel alone and lonely in this country. Sometimes it feels like you’re struggling all by yourself. Nobody cares, it’s every man for himself.”*

Several women reported having difficulty trusting people. They feared that they might unknowingly share their immigration status with someone with malicious intent who might report them to immigration authorities. Thus, secrecy was of utmost importance. Being secretive about their status and their challenges, however, had negative implications for women as it often meant that they sometimes did not feel comfortable reaching out to members of their community for assistance and for helpful information that would connect them to resources.

Women’s experiences of isolation were also a result of the discrimination they faced. Discrimination as a central part of women’s experiences in the U.S. has been discussed at length in a different manuscript. However, it is important to note that women faced discrimination in different facets of their lives and as a result of their identity of being Black, undocumented and an immigrant. This identity complicated women’s realities in the U.S. and contributed to their experiences of isolation and marginalization.

### 3.2. Mental Health Concerns

The many stressors that women coped with had implications on their emotional well-being. Women in the study expressed concerns about their emotional well-being and their mental health. Emotional well-being was being impacted by socio-political context, immigration status, and life as an immigrant. This main theme is divided into two subthemes-feeling sad and lack of peace.

#### 3.2.1. Feeling Sad/Depressed

Women reported feeling of sadness when confronted with their realities. One participant described how feelings of isolation caused her a lot of anxiety and worsened her depression. Over half of the participants reported recurrent feelings of sadness and depression over the course of their stay in the U.S. Sadness was attributed to economic challenges, isolation and the uncertainty women faced. Because women awaiting an immigration proceeding decision could not leave the country, several participants had not seen their family in years. Women described feeling sad due to missing their loved ones (son, parents, mother). One participant stated:

*“Everyone just seem to do their own thing. That sense of community is not really there. Things are hard for me now but the first few months were the hardest … you know adjusting to this new place. I was just sad and depressed all the time. I questioned my decision to come here every day. I don’t know. It was hard. Just so many different challenges. It’s like I left [name of country omitted to maintain confidentiality] with a specific idea of what America would be like. But then when I got here, I got the shock of my life.”*

Other participants’ sadness and feelings of depression stemmed from “feeling stuck” and “lacking a sense of purpose”. One participant expressed this sentiment saliently:

*“There is so much tension in the country right now … you don’t know who’s on your side and who’s not. You’re not sure who to confide in … and I don’t know what the future holds. I feel like I am wasting my potential. It really gets me down a lot but I try my best to keep my spirits up”*

This emotion of feeling unfulfilled was also described by a participant who had earned a Master’s degree in her field. After graduation, she was confronted with the reality that without work authorization, she could not get a job that matched her new qualifications, nor could she pursue a doctoral degree, as she had hoped to. As a result, she struggled with finding a sense of purpose, considering her circumstances. This led her to spiral into depression. She stated:

*“It got to a point that they actually wanted to put on medications. Because of everything, I would be happy one moment and sad the next. It was so bad that they were going to put me on prozac because of my mood and the things I was going through.”*

This participant’s situation was quite common as women with post-secondary education were often underemployed and not working in their field, using the degrees they had in fact earned. Women reported that their situation and the challenges they faced often made them tearful. Even talking about their experiences invoked emotion as the interviewer had to pause at least three interviews to provide emotional support to participants. Yet, due to issue of access and lack of trust of healthcare providers, only one of the women interviewed had discussed her symptoms with a healthcare professional. Other women coped with their sadness and viewed it as a normal part of their experiences.

#### 3.2.2. Lack of Peace

Women also described feelings of anxiety and fear. These were largely attributed to the many uncertainties with which they had to cope. They worried about being detected and/or deported and about the outcome of their pending immigration proceedings. The fear and anxiety that women experienced was a central part of their being undocumented and it had implications for the women’s ability to conduct their daily activities. Due to the fear of discovery, women had to be strategic about what type of spaces they accessed and when.

Generally, participants lived in a state of constant fear. They were scared to drive, and afraid to step out of their homes. Their fear even threatened their ability to seek healthcare. Women felt as if they were a target and they might be approached by immigration authorities at any time. This fear was heightened by women’s understanding of the current administration’s efforts to increase the deportation of undocumented immigrants. One participant’s description of her perception of the current sociopolitical environment embodied the women’s emotional state. She stated:

*“When Obama was the president, I felt this sense of normalcy. The fear I feel now was not as pronounced. But then with this administration, you realize that your whole life could be changed with a flick of a pen, basically.”*

Women reported that the anxiety and fear they felt varied in intensity depending on their situation at the time and the sociopolitical climate. Participants who had been in the U.S. for longer periods of time were more likely to report varying level of anxiety that reflected the changing policies and practices. Additionally, women with a longer stay in the U.S. were more likely to have attempted to regularize their status in the U.S. at some point. There were thus times when women did experience a stronger sense of security and less anxiety. For instance, one participant discussed being able to get work authorization after being sponsored by a spouse through which she could submit a petition to change her status. Obtaining work authorization enabled her to seek and gain formal, stable employment and feel more secure as she then believed she would soon be regularizing her status. However, her situation became complicated when her spouse decided against proceeding with the process. Consequently, the participant remained on the radar of immigration authorities and regained a heightened sense of vulnerability to deportation. This illustrates the dynamic nature of women’s experiences with feelings of anxiety and fear.

### 3.3. Coping

Women found ways to cope with their complex realities. The coping strategies women described often fell into two categories: (1) finding trusted people and (2) relying on religion/faith.

#### 3.3.1. Finding Trusted People

Social support was an essential component of women’s continued survival and coping. Prior to emigrating from their native country, most women had an acquaintance, relative or a friend who already resided in the U.S. Over time, women were able to expand their social circle to include other individuals in whom they could confide. The building of social networks appeared to occur in a snowball form where women would be linked to other individuals by known friends. One participant shared her experience in identifying individuals whom she could confide in:

*“So I’ve been trying to branch out like by doing this interview. Just trying to being more open with my friends with my family so that I can talk about my experiences and what I have been through because of my status. When I’m in immigrant spaces with other immigrants who would understand then I feel more comfortable. But that does not mean I walk around telling everyone about my status either. It means I’m more comfortable talking about my experiences with the people I trust in my circle. So for me, the people I tell are other black people who are also undocumented versus other immigrants who might hold certain opinions about you based on your status.”*

Women’s social network served as an outlet for them to share their struggles with others with who they believed they could somehow identify and relate and who in turn could understand their experiences. They discussed identifying other undocumented African immigrants who they trusted through referrals from another friend, relatives or other benefactors. Beyond that, social networks of friends, families, and faith communities also served the purpose of connecting them to resources and helping them navigate the many barriers they faced. For the women interviewed who mostly identified as practicing Christians, finding a religious or faith community occurred shortly after their arrival to the U.S. Some of the key individuals that women considered confidantes were religious leaders such as a pastors or other key figures within their faith communities. One participant who was detained by immigration officials described how her faith community and local community rallied around her and helped her raise the money needed to hire a lawyer who would represent her. Women were also able to get connected with resources without necessarily sharing their immigration status. For instance, another participant discussed reaching out to her church for financial assistance when she could not afford to pay her rent. She informed them that she was having financial difficulty but did not want to disclose her immigration status.

Women’s social support networks also comprised of loved ones in their home country. Networks in their home country were particularly important as forming relationships with people in the U.S. was complicated by their fear of betrayal and not knowing who to trust. Participants reported calling friends and family in the home countries often to seek encouragement and to share their burdens. Beyond providing emotional support, social networks in women’s home countries also provided them with instrumental support. Participants reported having their loved ones from their home countries send them food items and medications.

#### 3.3.2. Relying on Religion/Faith

Religion was central to women’s experiences of being resilient in the face of their challenges and complex realities. Religion gave women hope and enabled them to have faith that their situation would change. Religiosity was particularly important for one participant who was detained and separated from her family. While she was at the detention center, she reported praying and fasting as a coping mechanism while detained. Another participant shared how her faith helped through a dark time:

*“I would say Jesus. I just had to pray. And even with prayer, it was still hard. Honestly, it was definitely hard. My reality was that everybody was out doing their own thing. And you start comparing yourself to other people. You see other people doing things and accomplishing things that you can’t. And you realize that nothing makes sense. It just felt that I just kept going deeper and deeper into depression. I just thank God for my friends and my family. They understood and were able to support me when I was moody.”*

From their religion and faith, women found the strength to persevere. For one participant, her religious affiliation and faith community helped give her the sense of purpose that she desperately needed. She shared her experiences volunteering with a church group and how that experience gave her a different outlook at a time when she was battling depression.

*“Ummm I think it’s a sense of purpose. It had to do with finding a sense of purpose. I got a volunteer position where I was able to like ... it was a missionary type volunteer activity where I was able to take the youth at my church and mentor people. That was the purpose I needed. Just something to remind me that I am important. You know? It got to a point where it was hard for me ... why am I here? What’s the purpose? Like what sense does it make? Why am I wasting my time on this earth?”*

Religion also provided a framework through which women could make meaning of their often-confusing realities. Women developed positive appraisals of their situations and attempted to describe how their experiences contributed to their growth in some way in spite of the vulnerability, uncertainty and isolation. They often framed their current hardship as a phase they believed would eventually end at some point while comparing their current challenges to past challenges they had already overcome through their faith. Despite the challenges that women faced, none of the participants expressed a persistent desire to return to their home country at the time of the interview, even though some women did express having intermittent episodes of feeling regret or wanting to return. They thus perceived their current phase as transitionary. Their belief that this phase of their lives was just another challenge that would pass, provided them with some degree of comfort.

## 4. Discussion

This qualitative descriptive study examined the stressors, mental health concerns and coping strategies of undocumented African women in the U.S. Our findings reveal that undocumented African migrant women experience significant stressors that have important implications on mental health. From a postcolonial feminist perspective, our analysis foregrounds how women’s challenges and resultant mental health symptoms occur within a given sociopolitical context that impedes access to services and resources, resulting in increased vulnerability and marginalization. Our use of a postcolonial feminist lens also supported the harnessing of knowledge on the agency of undocumented African migrant women. Despite the challenges they experienced, these women nevertheless resisted the impact of their hostile context and demonstrated resilience by relying on social support and their faith.

Similar to previous studies on the mental health needs of undocumented immigrants in the U.S., the women interviewed reported anxiety and sadness [27], with some women in the study describing symptoms of severe depression. Despite experiencing these symptoms, most women had not had the opportunity to discuss their symptoms with a healthcare provider. This is an expected finding as undocumented immigrants experience complex barriers to healthcare access [45]. Difficulty accessing mental health services has serious implications for women’s health and well-being. Chronic stress and resultant psychological distress impacts health outcomes as uncontrolled anxiety has been shown, for instance, to increase the risk for cardiovascular disease [46].

Even when undocumented patients are able to access healthcare, getting specialty care such as mental health services may be challenging. Considering that undocumented immigrants are likely to seek healthcare from primary care safety-net clinics, safety-net healthcare providers should be equipped, and resources made available to attend to their mental health needs. Within the healthcare setting, assessing the mental health needs of undocumented immigrants requires culturally appropriate tools. There is a need for healthcare providers to have basic knowledge about the challenges that immigrants, and particularly undocumented immigrants, face in the current sociopolitical climate. Lack of awareness or understanding of the realities of undocumented people might pose a barrier to effective screening and interventions that would address their mental health needs. This is especially important for providers who cater for large immigrant communities. It is also vital that healthcare providers employ culturally appropriate screening tools that account for potential variations in the conceptualization of mental health symptoms among different immigrant groups. For instance, compared to the dominant Western notion of duality of physical and emotional parts of the body, some immigrants might somaticize emotional distress and report physical symptoms [47].

The findings presented in this paper are novel for several reasons. First, this study adds a critical, analytical dimension that is often missing in existing literature linking undocumented migrants’ experiences to their context. Our findings demonstrate the interrelatedness of women’s given sociopolitical context and their emotional well-being, a phenomenon which is supported in current literature. In a study with Latino immigrants in the U.S., Hatzenbuehler et al. [48] found an association between undocumented immigrants’ residing in states with exclusionary immigration policy and increased rates of poor mental health days. Similarly, the women in the study expressed change in severity of distress and feelings of marginalization across varying temporal contexts. The current hostile sociopolitical context meant that their daily lives were characterized by challenges that affected their sense of security and undermined economic and social capital. Second, this study also adds the narratives of Black, African migrants, which is missing in existing literature on undocumented immigrants. We found that the Black, African women we interviewed generally lacked a sense of belonging. They felt as if most immigrants’ rights advocacy and support efforts focused on the plight of undocumented Latino/a immigrants. They also felt excluded from community organizations and advocacy efforts that appeared to only champion the cause of African Americans. Hence, there appeared to be a lack of supportive spaces to make them feel less isolated.

Women’s reliance on faith and religion to cope offers an opportunity for faith communities to be intentional about helping immigrant women feel less isolated. This can be accomplished by developing programs and avenues through faith communities where women can build social networks and become connected to helpful resources. Low social support in immigrants has been associated with higher risk of reporting psychological distress [49]. Given that social support has been shown to be an effective coping strategy for individuals with mental health symptoms [50], support groups of immigrants experiencing the same challenges could be therapeutic for women and could help them develop meaningful relationships with other immigrants and even non-immigrants.

Considering the strong ties to faith communities the women reported in the study, religious organizations can serve as a critical access point for safety-net providers to reach undocumented women as interventions directed through their faith communities could increase the ease of access. Partnership between faith-based organizations and healthcare professional to improve the health outcomes within underserved communities is not a novel idea. This approach has been applied to increasing cervical cancer screening and target obesity, in Appalachian community and African American communities, respectively [51,52]. Other specific examples of such initiatives include a church-based health education and community-based outreach to vulnerable population using faith-based organizations [53].

A fundamental driver of women’s hardships and the associated psychological distress is their immigration status. It has been established that the type of chronic stress that typifies women’s daily lives leads to anxiety and depression [54]. The findings of this current study are relevant given the inaction of the legislative branch to introduce legislation on immigration reform. Evidently, there is a need for an immigration reform that grants immigrants with precarious status, protected and permanent status. Doing so, will decrease fear in immigrant communities, grant them access to basic resources and formal employment and enable them to come out of the shadows. Lastly, there is a need for further research on the mental health outcomes of undocumented immigrant and the development of culturally appropriate interventions that effectively respond to their needs. Current research is scant and is lacking in sample diversity. Future studies should examine how women’s experiences differ by region of residence. Studies should also report on the longitudinal mental health outcomes of undocumented women.

There were a few limitations to the study. Firstly, the study included only 24 women. Though this was a relatively small sample, theoretical saturation was achieved. Secondly, most of the women interviewed were from the Midwestern part of the U.S. Due to the potentially varying sociopolitical climate across regions of the U.S., we may not have captured the more nuanced experiences that characterize the lives of undocumented African women living in other regions of the country. Nevertheless, we do believe that the patterns related to undocumented women’s experiences that we have identified here would essentially be similar across geographic regions of the U.S. Lastly, four interviews were conducted via phone. As a result, the researcher was unable to note nonverbal cues and make perceptual observations. However, data from in-person interviews corroborated findings obtained from telephone interviews.

## 5. Conclusions

The challenges that undocumented African women face increase their risk of psychological distress. Given that the women’s distress and precarity will likely persist while they lack a recognized immigrant status, effective coping strategies and access to mental health care are essential in helping them manage their complex realities. Through the women’s narratives reported in this paper, healthcare providers, faith communities and other allies can gain insight into what resources would be most helpful to women in alleviating some of the stressors they experience. For the women interviewed, social support provided an outlet for them to gain support and obtain information, which was useful in helping them problem-solve some of their challenges. Hence, effective interventions for undocumented migrant women should incorporate social support while simultaneously addressing stressors associated with material hardship. For instance, the women in the study may have strongly benefitted from having access to a safe space where all migrants— regardless of immigration status or nationality—could receive instrumental and emotional support. Additionally, access to mental health care was lacking for the women interviewed. Though undocumented immigrant women may not disclose their immigration status, it is essential that healthcare providers proactively assess their immigrant patients’ psychological health during healthcare encounters. This is a critical consideration in the current sociopolitical context that has clearly heightened undocumented immigrants’ feelings of marginalization and vulnerability.

## Figures and Tables

**Table 1:** Demographic data for women interviewed (n = 24).

Variable	Mean
Age (23–55 y)	35.69
Length of Stay in U.S. (2–22 y)	11.52
	**n (%)**
Marital Status	
Married	7 (29%)
Widowed	1 (4%)
Divorced	1 (4%)
Separated	3 (13%)
Never married	12 (50%)
Children	
Yes	14 (58%)
No	10 (42%)
Region of Residence in U.S.	
Midwest	21 (88%)
South	2 (8%)
East	1 (4%)
Employment Status	
Full time (40 h a week)	9 (38%)
Part time (<40 h a week)	1 (4%)
Casual worker (work when work is available)	7 (29%)
Unemployed	7 (29%)
Highest Level of Education	
High school	4 (17%)
Some college credits	2 (8%)
Associates degree	7 (29%)
Bachelor’s degree	8 (33%)
Graduate degree	3 (13%)
Annual Household Income	
Less than $10,000	4 (17%)
$10,000 to $19,999	9 (38%)
$20,000 to $29,999	5 (21%)
$30,000 to $39,999	4 (17%)
$40,000 to $49,999	1 (4%)
$50,000 or more	1 (4%)

## Appendix A. Interview Guide

1. How did you move to the United States?
  - How were things for you after moving here? What challenges did you face?
2. How does it feel being an undocumented African woman in the United States?
  - What challenges do you face?
  - How do you overcome these challenges?
3. What is your current job? Is it different from what you did in your country of origin?
4. Can you tell me about the conditions under which you work?
5. Where do you go when you are not feeling well?
  - Where do your children go when they are not feeling well?
6. How does being undocumented affect your ability to get health care?
7. Tell me about your experiences using the healthcare system in the United States.
  - Could you tell me about a time when you had a good experience while trying to get health care? How were treated by the health care workers?
  - Could you tell me about a time when you had a bad experience while trying to get health care? How were treated by the health care workers?
8. What concerns you have about your health? Your emotional well-being?
9. What do you do when you are feeling stressed?
10. Do you currently have any concerns about your safety?
  - What would you do if you felt like you were in danger or if you were a victim of a crime?
